# Surface Rendering of External Genitalia of a Fetus at the 32nd Week of Gestation Affected by Partial Androgen Insensitivity Syndrome

**DOI:** 10.1155/2013/325714

**Published:** 2013-08-19

**Authors:** Vincenzo Mazza, Emma Bertucci, Silvia Latella, Carlotta Cani, Pierluca Ceccarelli, Lorenzo Iughetti, Fulvia Baldinotti, Antonio Percesepe

**Affiliations:** ^1^Prenatal Medicine Unit, Policlinico Modena, Via Largo Del Pozzo 71, 41124 Modena, Italy; ^2^Unit of Pediatrics Surgery, Department of Mother & Child, Modena Hospital, Modena and Reggio Emilia University, Via Del Pozzo 71, 41110 Modena, Italy; ^3^Unit of Pediatrics, Department of Mother & Child, Modena Hospital, Modena and Reggio Emilia University, Via Del Pozzo 71, 41110 Modena, Italy; ^4^Unit of Cytogenetics and Molecular Genetics, Department of Mother & Child, Pisa University, Italy; ^5^Unit of Medical Genetics, Department of Mother & Child, Modena Hospital, Modena and Reggio Emilia University, Via Del Pozzo 71, 41110 Modena, Italy

## Abstract

*Objectives*. To demonstrate the feasibility of the prenatal diagnosis of partial androgen insensitivity syndrome by 3D-4D ultrasound. *Methods*. To report prenatal diagnosis of partial androgen insensitivity syndrome at 32nd week of gestation by 3D-4D ultrasound in a fetus with a 46XY karyotype, testing negative to the mutation analysis of SRY gene and the 5**α**-reductase 2 gene (SRD5A2). *Results*. 3D-4D surface rendering allows the detection of external and internal genital malformations and can address the prenatal diagnosis of PAIS and can exclude associated complications. *Conclusions*. Prenatal diagnosis of PAIS allows an adequate parental counseling and an early optimal management of the condition, not only for the psychological and social reflections but also for the avoidance of complications and postnatal morbidity due to misdiagnosis or delays in the treatment of the genital ambiguity.

Androgen insensitivity syndrome (AIS; MIM no. 300068) represents the most common identifiable cause of male pseudohermaphroditism, accounting for as many as 1 : 20.000 births [1]. The disease is transmitted in an X-linked recessive fashion and is featured by an abnormal development of both internal and external genital structures in 46 XY individuals. The associated phenotypes range from male infertility or undervirilization (partial androgen insensitivity syndrome (PAIS) (grade 1)), to completely normal female external genitalia (complete androgen insensitivity syndrome (CAIS) (grade 6/7)), in rank of increasing severity of androgens resistance [2]. PAIS is diagnosed when gonadotropins and testosterone are normal, but the physiological androgen response in target tissues is absent or decreased, due to mutations in the androgen receptor (AR) gene, located in Xq12 [1, 3]. We report a case of a 35-year-old healthy pregnant woman, primigravida at the 32nd week of gestation, that was referred to our prenatal diagnosis unit for a mild polyhydramnios. Chorionic villus sampling (CVS) had been previously performed for maternal age at the 11th week, revealing a normal 46 XY karyotype. Routine prenatal ultrasonographic examination performed in another center at the 20th week and 30th week of gestation revealed a normal fetus with no mention of the external genitalia. We performed 2D and 4D ultrasonographic (US) examinations using Voluson E8 (GE Healthcare, Milwaukee, WI, USA) with a volumetric 4–8 MHz transducer, observing a normal amount of amniotic fluid and revealing the presence of female/ambiguous genitalia in an otherwise normal fetal anatomy. The 4D surface rendering showed typical female genitalia with mildly oedematous labia majora and clitoromegaly (Figure 1(a)). The fetal uterus was not visualized with both conventional 2D US and 3D US with VCI software in A-plane.

To clarify the discrepancy between the male karyotype and the female genital phenotype, an amniocentesis was performed and the fetal DNA extracted from the cultured amniocytes was tested by direct sequencing for mutations in the SRY gene and in the 5*α*-reductase 2 gene (SRD5A2) to exclude other causes of hermaphroditism or pseudohermaphroditism. Both analyses did not reveal any mutation. 

Therefore, the presence of female external genitalia without the uterus, in association with normal SRY and SRD5A2 genes, led us to the diagnostic hypothesis of (complete or partial androgen insensitivity syndrome) (CAIS/PAIS), that was tested through the sequence analysis of the AR gene which did not show any mutation. A multidisciplinary counseling session was scheduled with the parents to address the issue of the female sex assignment at birth. Delivery was at the 39th gestational week. The female external genitalia with clitoromegaly at birth are shown in Figure 1(b); moreover, the clinical inspection of the external genitalia revealed palpable gonads bilaterally in the labioscrotal folds. The hormonal blood profile detected normal concentrations of testosterone (79 ng/dL) and androgens (DHEAS 7.5 mcgr/mL, androstenedione 209.4 ng/dL) for age and male sex and also after hCG test (testosterone 146 ng/dL, DHEAS 7.5 mcgr/mL, and androstenedione 126.4 ng/dL). Urethrocystoscopy showed the presence of a low urogenital sinus, corresponding to the 4th degree of PAIS, defined as severely limited masculinization with phallic structure intermediate between clitoris and penis and urogenital sinus with perineal orifice and labioscrotal folds [2]. Videolaparoscopy revealed the complete absence of internal female genital structures in the Douglas cavity and the presence of internal spermatic blood vessels and deferent ducts ending bilaterally into the peritoneal vaginal duct with an opened internal orifice. In order to achieve the complete female phenotype, the newborn underwent surgical treatment of perineal vulvovaginoplasty, clitoroplasty, and gonadectomy (Figure 1(c)). Pathological examination demonstrated that the gonads were represented by normal testicles with normal epididymis and deferent ducts.

In the present report, we show how the prenatal diagnosis of PAIS allows an adequate parental counseling and an optimal management of the condition, not only for the relevant psychological and social implications of an early diagnosis but also for the reduction of the chance of complications and postnatal morbidity due to misdiagnosis or delays in the treatment of the genital ambiguity. 

The contribution of US in prenatal sex determination is well known, and its role in detecting sexual differentiation disorders has been previously established [4]. In addition, the limitations of standard 2D fetal US [5] have been overcome by the advent of 3D-4D US that has allowed the study of the anatomy of the internal genitalia [6, 7]. In particular, the exclusion of the presence of the fetal uterus at weeks 32–34 of pregnancy by 3D VCI in A-plane has been crucial for the prenatal diagnosis of the present case.

The mainstay of the diagnosis of PAIS is the presence of a 46 XY karyotype, the determination of the normality (baseline or after hCG stimulation) of testosterone and dihydrotestosterone levels (in order to exclude defects in testosterone biosynthesis or 5*α*-reductase 2 deficiency), and the confirmation of the diagnosis through the identification of a molecular defect in AR gene. In our case, direct sequencing of all of the 8 exons of the AR gene did not detect any mutation, consistent with the mutation rate of 28% to 73% for the incomplete, milder phenotypes [1].

In conclusion, 3D-4D US and surface rendering have demonstrated their power in the detection of external and internal genital malformations, and in association with the molecular exclusion of the main causes of hermaphroditism and pseudohermaphroditism they have addressed the prenatal diagnosis of PAIS, although it is not confirmed by the finding of the mutation in the AR gene. Also the ability to exclude associated complications (i.e., urological) has given the possibility of a reassuring prenatal counseling session about the excellent prognosis of the condition, contributing to improve the quality of life and health for both the child and the parents.

## Figures and Tables

**Figure 1:**
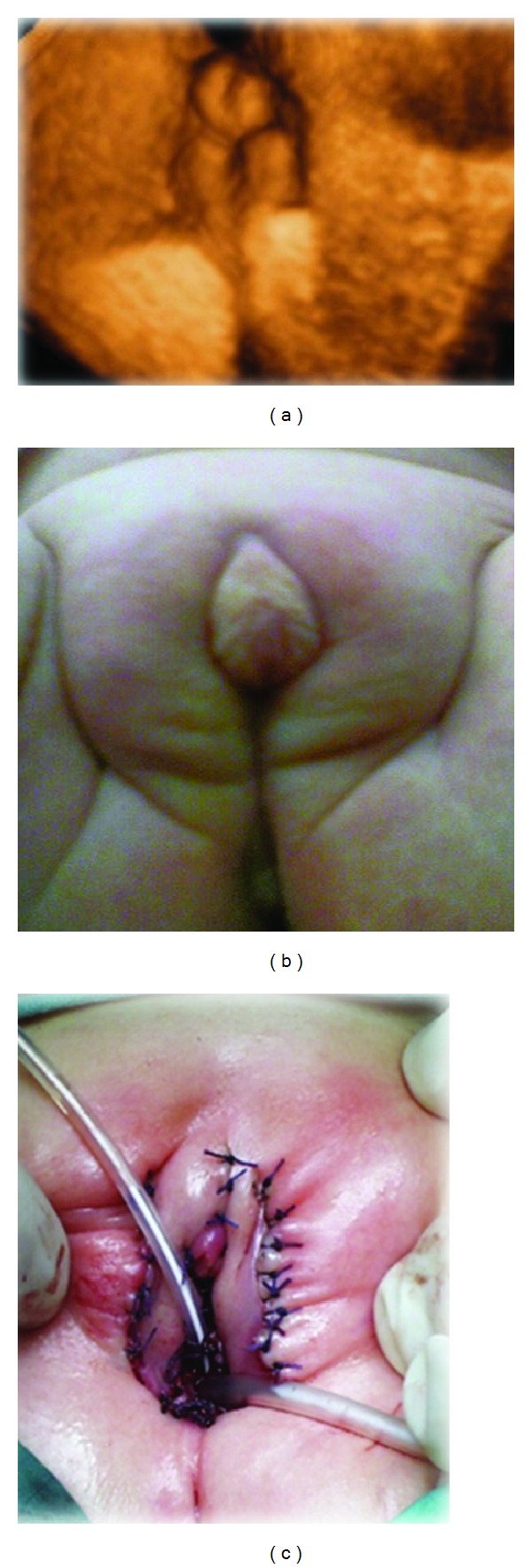
(a) 4D surface rendering female/ambiguous external genitalia; (b) female genitals with oedematous labia majora and mild clitoromegaly; (c) perineal vulvovaginoplasty, clitoroplasty, and gonadectomy.
